# Recent Advances in Additive Manufacturing of Soft Magnetic Materials: A Review

**DOI:** 10.3390/ma16165610

**Published:** 2023-08-13

**Authors:** Bryan Ramiro Rodriguez-Vargas, Giulia Stornelli, Paolo Folgarait, Maria Rita Ridolfi, Argelia Fabiola Miranda Pérez, Andrea Di Schino

**Affiliations:** 1 Dipartimento di Ingegneria, Università degli Studi di Perugia, Via G. Duranti 93, 06125 Perugia, Italy; bryanramiro.rodriguezvargas@studenti.unipg.it (B.R.R.-V.); giulia.stornelli@unipg.it (G.S.); 2 Seamthesis Srl, Via IV Novembre 156, 29122 Piacenza, Italy; paolo.folgarait@seamthesis.com (P.F.); mariarita.ridolfi@seamthesis.com (M.R.R.); 3 Department of Strategic Planning and Technology Management, Universidad Popular Autónoma del Estado de Puebla, 17 Sur, 901, Barrio de Santiago, Puebla 72410, Mexico

**Keywords:** additive manufacturing, soft magnetic materials, microstructure, magnetic properties

## Abstract

Additive manufacturing (AM) is an attractive set of processes that are being employed lately to process specific materials used in the fabrication of electrical machine components. This is because AM allows for the preservation or enhancement of their magnetic properties, which may be degraded or limited when manufactured using other traditional processes. Soft magnetic materials (SMMs), such as Fe–Si, Fe–Ni, Fe–Co, and soft magnetic composites (SMCs), are suitable materials for electrical machine additive manufacturing components due to their magnetic, thermal, mechanical, and electrical properties. In addition to these, it has been observed in the literature that other alloys, such as soft ferrites, are difficult to process due to their low magnetization and brittleness. However, thanks to additive manufacturing, it is possible to leverage their high electrical resistivity to make them alternative candidates for applications in electrical machine components. It is important to highlight the significant progress in the field of materials science, which has enabled the development of novel materials such as high-entropy alloys (HEAs). These alloys, due to their complex chemical composition, can exhibit soft magnetic properties. The aim of the present work is to provide a critical review of the state-of-the-art SMMs manufactured through different AM technologies. This review covers the influence of these technologies on microstructural changes, mechanical strengths, post-processing, and magnetic parameters such as saturation magnetization (M_S_), coercivity (H_C_), remanence (B_r_), relative permeability (M_r_), electrical resistivity (r), and thermal conductivity (k).

## 1. Introduction

In recent years, efficiency and sustainability in the energy sector (such as the oil and gas industry, aerospace, and automotive sector, specifically electric vehicle production) [1,2,3,4,5,6,7], along with the demand for a reduction in pollutant emissions, have driven the advancement of materials for such purposes. These advancements extend to manufacturing methods as well, most prominently additive manufacturing (AM), driven by the imperative to curtail pollutant emissions.

The AM process, known as “3D printing,” is an innovative manufacturing process that allows the generation of components, layer by layer, directly from a Computer-Aided Design (CAD) model. Among the various qualities of this technology, there is the ability to produce complex geometries, which has generated considerable interest and development within various industries [8,9,10,11,12,13].

AM has facilitated the rapid creation of prototypes and a reduction in material waste. This can be justified from a comparative standpoint by considering the value of the metal in additive manufacturing versus traditional machining, considering factors such as product performance, time savings, and production costs. As a result, AM is becoming increasingly competitive in terms of speed and cost. Consequently, it has emerged as an appealing option for various industries seeking efficient and cost-effective production methods, contributing to its rapid growth and implementation [14,15,16,17,18]. In addition to cost savings, AM has the potential to generate substantial energy savings in the final product compared to those manufactured using traditional machining methods. This is particularly relevant in industries that require lightweight electric machines, including transportation, renewable energies, passenger aircraft, and naval applications. Conventional manufacturing techniques often impose limitations on lightweight designs, but as mentioned earlier, AM has introduced unprecedented design freedom. New opportunities have arisen for the manufacturing and design of lightweight electric machines, which can enhance the efficiency and performance of these applications [19,20,21,22,23,24,25,26,27]. This requirement is particularly critical in several industrial sectors that consume a significant portion of the total electricity, with electric motors and machinery accounting for at least 65% of this consumption. For instance, an efficiency improvement of just 1% could result in a reduction in several million metric tons of hazardous emissions [28,29]. Therefore, by exploiting unconventional manufacturing techniques, such as AM, the researchers aim to overcome the limits of traditional processes and promote significant contributions to energy savings and environmental sustainability.

Another notable feature of this manufacturing process is the wide range of materials that can be used in the various components required by the industry, including polymers, biopolymers, metals, and composites, among others. This extensive material selection opens new possibilities for innovation and customization in various industrial applications. This is particularly relevant in the case of soft magnetic materials (SMMs) employed in electromagnetic devices used for energy conversion and generation [30,31,32,33].

SMMs with an iron base alloy with the addition of silicon (Si), nickel (Ni), and/or cobalt (Co) exhibit high saturation induction, low coercivity, high permeability, and low core loss. As a result, SMMs find widespread application in electric motors, generators, and transformers, ensuring optimal energy conversion and facilitating energy generation and transmission [28,34,35].

The importance of AM in the processing of soft magnetic materials can be understood by considering their conventional manufacturing methods. Traditionally, these materials have been fabricated in various forms, such as foils, plates, sheets, and bars, following a series of steps known as “ingot metallurgy practices,” involving sequential processes of melting, iterative thermomechanical forming, and annealing treatments. Casting is the initial step, where the molten alloy is poured into molds to form ingots. These ingots are then subjected to thermomechanical forming processes, such as rolling, forging, or extrusion, to refine the microstructure and improve the mechanical properties of the alloys. After that, the alloys undergo annealing treatments to promote recrystallization and grain growth [28,36,37,38]. Such multi-step processing techniques, involved in ingot metallurgy practices, play a significant role in the magnetic behavior of these alloys. However, during conventional manufacturing processes, some solid-state disorder–order phases tend to form, reducing the workability of soft magnetic alloys and limiting the optimization of the chemical composition of the alloy [39,40,41]. For example, in high-silicon Fe–Si alloys, there is a tendency during cooling to form phases with oriented structures through lattice rearrangement into B2 and D03 phases [42,43]. This is known to increase material brittleness to the extent of limiting workability at low temperatures [39].

In this regard, by exploring new emerging manufacturing methods, such as additive manufacturing, it is possible to integrate or replace traditional processing techniques. This opens significant opportunities for the development of SMMs with improved performance and processability suitable for a wide range of electromagnetic applications, leading to improved efficiency.

AM processes, such as Laser Powder Bed Fusion (L-PBF), Selective Laser Melting (SLM), Directed Energy Deposition (DED), and Spark Plasma Sintering (SPS), among others, offer significant advantages in the production of various components, including the ferromagnetic core for the electrical motors. For Fe–Si alloys, additive technology provides an alternative to preprocessing variants with high silicon content. Indeed, the rapid cooling rates achieved during the laser melting process prevent the occurrence of the usual ordered–disordered phase transition in Fe–Si steels. Furthermore, in the conventional Fe–Si rolling and stacking process, the sheet sizes are limited by the available rolling mills as well as the chemical composition of the alloy itself, resulting in a limited ability to create complex geometries for the magnetic flux path. Additive technology offers greater design freedom, enabling the production of parts with complex geometries and intricate internal structures. This allows for the optimization of the magnetic flux path and, consequently, the electromagnetic performance of the device, leading to higher efficiency and improved energy conversion [44,45,46,47]. This technology has also led to the development of Fe–Co and Fe–Ni alloys, allowing precise control of the resulting magnetic properties, including high saturation magnetization and low iron losses [46,48,49]. Materials such as soft ferrites, which are unsuitable for use in rotors due to their brittleness and low magnetization, could be processed thanks to advancements in additive manufacturing. This allows for their utilization based on their high electrical resistance, employing them as insulating materials in electric cores [50]. Soft magnetic composites (SMCs) are novel materials characterized by low core loss, high saturation magnetization, and high electrical resistivity. They are composed of magnetic particles coated with insulation, allowing for the creation of three-dimensional flux paths. However, obtaining these geometries is limited by the capacity of the compaction process to achieve uniform pressure. As mentioned earlier, the capability of AM to fabricate complex geometries has facilitated the use and development of these materials. An example of this is the study carried out on the AM processing of FINEMET^®^, which is a trademark of a SMC with extremely high permeability and significantly high electrical resistivity. The utilization of techniques such as laser-engineered net shaping (LENS™) for its processing has led to the attainment of coercivity values exceeding 2000 A per meter [51,52,53]. By manipulating the chemical composition of materials and matching operating parameters (energy input, scanning speed, heat treatment, etc.), AM provides opportunities to tailor the magnetic characteristics of components, optimizing their performance in specific applications [54,55,56,57,58].

**Figure 1:**
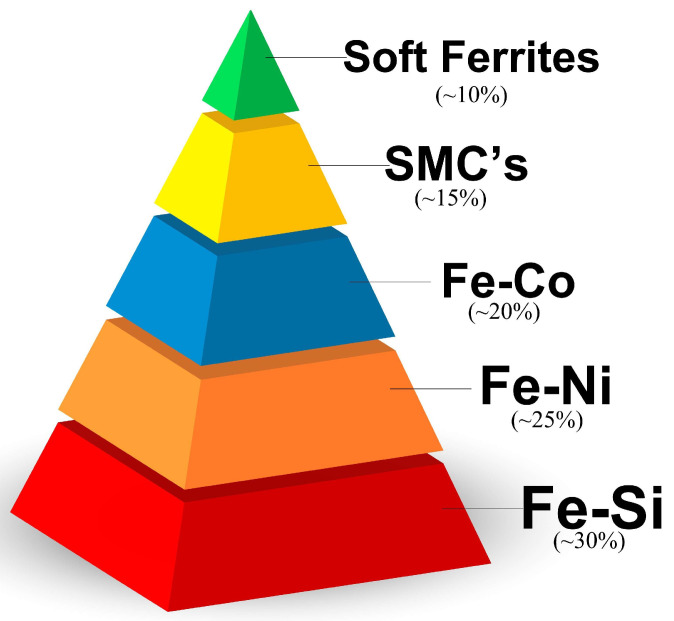
Summary of the soft magnetic materials utilized in the reviewed articles.

The state-of-the-art review of additive manufacturing in magnetic materials for this research covered a period of investigation up to the year 2023. Figure 1 illustrates the various alloys used as soft magnetic materials that were covered in this work, along with the semi-quantitative percentage of papers found in the literature review. It is noteworthy that Fe–Si and Fe–Ni alloys maintain their significance in the manufacturing of electromechanical devices for energy conversion and generation. However, SMCs, despite being relatively recent materials compared to others, exhibit a clear increasing trend in publications on the topic. It is important to mention that at the end of this review, special emphasis is placed on the immediate future of SMMs, which includes the exploration of high-entropy alloys (HEAs). These alloys are considered magnetic materials that, based on the control and manipulation of their chemical composition, can exhibit soft or hard magnetic properties depending on their application.

The importance of studying these soft magnetic materials lies in the impact of their favorable magnetic properties, which allow them to respond quickly to external magnetic fields and minimize energy losses due to magnetic hysteresis. Therefore, electromechanical devices that utilize soft magnetic materials can achieve high efficiency and performance. The objective of this document is to provide a summary of the recent advancements in utilizing soft magnetic materials in AM for the design of electrical machines. It will explore the advantages and limitations of processability for each alloy, as well as the AM technologies employed. This comprehensive overview aims to contribute to the advancement of this field by inspiring further research and fostering innovation.

## 2. Overview of Soft Magnetic Materials

**Figure 2:**
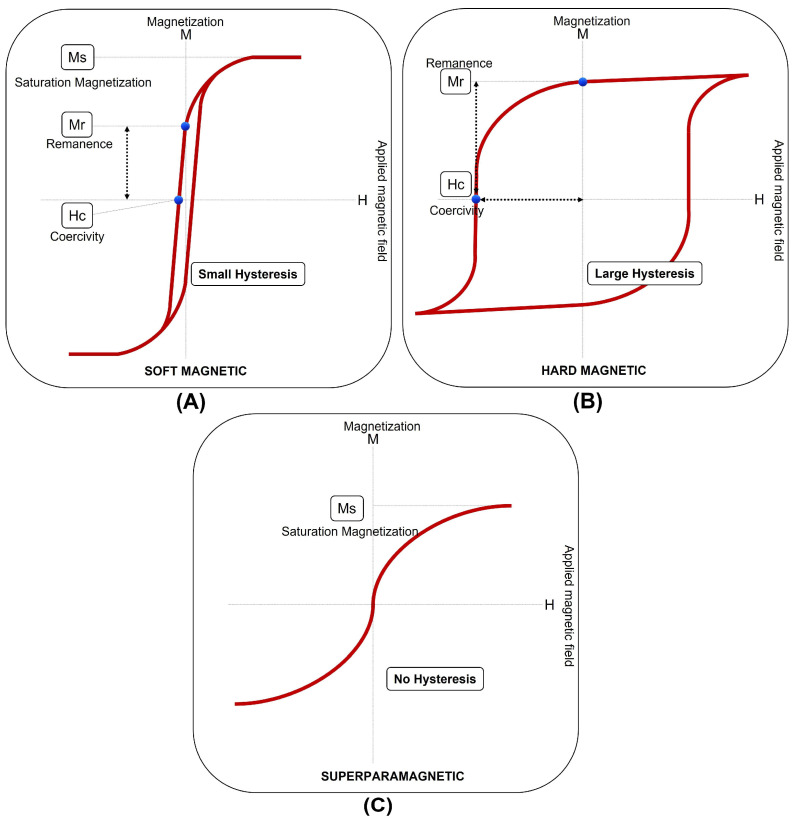
Magnetic behavior of different classes of ferromagnetic materials: (**A**) soft magnetic materials; (**B**) hard magnetic materials; and (**C**) superparamagnetic materials.

The term “magnetic materials” is commonly used to refer to ferromagnetic materials that exhibit strong attraction to magnets due to their spontaneous magnetization. These materials possess inherent magnetic properties that allow them to be easily magnetized and demagnetized [56,57,58,59,60,61]. This can be classified into three distinct groups: soft-magnetic materials, hard-magnetic materials, and superparamagnetic materials [61]. Figure 2 illustrates the qualitative distinctions among these groups of magnetic materials based on their magnetization curves. In the subsequent lines, a brief explanation of each of these materials will be provided to explain their differences. However, given the primary focus of this research, the emphasis of the following paragraphs and consecutive sections will be on soft magnetic materials.

Soft magnetic materials (SMMs; Figure 2A) are called “soft” because they can be magnetized with low excitation, reach high values of magnetic induction, possess high magnetic permeability, and are easily magnetized and demagnetized. They exhibit a magnetization curve that saturates at relatively low magnetic fields. SMMs are commonly used in applications where efficient magnetic flux circulation and low energy losses are essential, such as transformers, electric motors, and magnetic sensors. According to the international standard, SMMs are those materials with coercivity less than 1 kA/m [62,63,64].

Hard-magnetic materials (Figure 2B), also known as “permanent magnets,” exhibit high coercivity and remanence, maintaining a significant portion of their magnetization even after the external magnetic field is removed. Hard-magnetic materials require high magnetic fields to reach saturation. These materials are used in devices requiring a stable and strong magnetic field, such as magnetic storage systems, magnetic resonance imaging (MRI) machines, and magnetic separators [65,66,67].

Superparamagnetic materials (Figure 2C) are characterized by their small particle size, typically on the nanoscale. Unlike hard-magnetic materials, superparamagnetic materials do not possess permanent magnetization. Instead, they exhibit a response to magnetic fields like SMMs. Superparamagnetic materials are widely used in biomedical applications, such as targeted drug delivery, MRI contrast agents, and magnetic hyperthermia for cancer therapy [68,69].

For the SMMs, there are two important parameters to consider: the remanent magnetization (M_r_) and the coercive field (H_C_). M_r_ represents the amount of residual magnetization in the material when the external magnetic field is removed. It indicates the material’s ability to preserve magnetization. H_C_ indicates the material’s resistance to demagnetization. It represents the magnetic field strength required to reverse the remanent magnetization and return the material to its demagnetized state. In addition, other significant parameters in magnetic materials include magnetic susceptibility (χ_m_) and saturation magnetization (M_S_). χ_m_ measures the extent to which a material can be magnetized in response to an applied magnetic field. It is proportional to the slope of the magnetization curve for magnetically isotropic materials. As the strength of the applied magnetic field increases, the material becomes more magnetized until it reaches saturation. Saturation occurs when all the magnetic domains of the material align with the applied field. Therefore, the induced magnetization reaches the M_S_ value, which represents the maximum magnetization that the material can achieve [50].

These parameters play a crucial role in characterizing and understanding the magnetic properties of materials. By measuring and analyzing these values, researchers can assess the material’s magnetic behavior and determine its suitability for various applications [61,70,71,72]. For example, Fe–Si and Fe–Ni alloys exhibit high magnetic susceptibility and saturation magnetization while maintaining low remanence and coercivity. This characteristic makes them easily magnetized, highly responsive to magnetic fields, but susceptible to demagnetization. Additionally, silicon steel alloys provide a well-balanced solution in terms of cost and performance for low-frequency applications of 50–60 Hz. However, for higher operation frequencies in the kHz/MHz range, SMCs such as amorphous and nanocrystalline materials are preferred due to their lower losses, despite their higher cost. In industries where high power densities are required, such as aerospace, Fe–Co alloys are commonly utilized, which possess excellent magnetic properties [73,74,75,76,77,78,79,80,81,82]. The response of SMMs to external magnetic fields is complex and primarily dependent on the chemical composition of the material as well as on the microstructure and texture. It is important to carefully consider the specific requirements and operating conditions of the application to select the most suitable soft magnetic material. Factors such as cost, performance, frequency range, and power density warrant careful consideration in this selection process [83,84,85,86].

As mentioned so far, soft magnetic materials exhibit a wide range of options, with performance varying significantly. Table 1 presents a general overview of different SMMs, such as Fe–Si, Fe–Ni, Fe–Co, SMCs, and soft ferrites, along with their relevant magnetic properties for industrial use, such as saturation magnetization (M_S_), relative permeability (μ_r_), and core resistivity (r). These materials are manufactured in various forms using additive manufacturing techniques such as L-PBF, SLM, DED, and/or SPS. The information provided in this table is highly useful for understanding the differences in properties and characteristics among various soft magnetic materials, enabling proper selection for the design and manufacturing of electromagnetic devices.

## 3. Fe–Si

Silicon steels (Fe–Si) have long been the standard industrial material for the manufacture of electric motor cores, generator cores, and transformer cores due to their remarkable electromagnetic properties and cost-effectiveness, making them highly competitive in the industry [39,50,137]. They are typically produced with a silicon content ranging from 2.0% to 7.0% wt. As the Si content increases, there is a substantial improvement in their soft magnetic characteristics. However, the material becomes more brittle, making it difficult to process using traditional methods [95,138]. This section is divided into two parts: the first one describes several recent studies on the processing of low-silicon Fe–Si alloys using different additive manufacturing processes. The second part focuses on high-silicon steels. In both parts, the aim is to illustrate the effect of process parameters such as energy source, scanning strategy, and post-processing techniques. These factors influence the microstructure, phase distribution, porosity, grain size, and texture of the materials, which in turn affect their magnetic and mechanical behavior.

**Figure 3:**
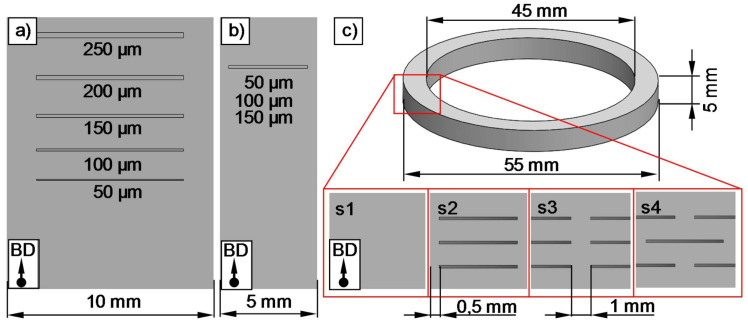
CAD-models of samples: (**a**) parallelepiped with five slits exhibiting constant thickness from 50 μm to 250 μm; (**b**) parallelepiped with one slit exhibiting constant thickness of 50 μm, 100 μm, or 150 μm; (**c**) ring specimen used for measuring the magnetic properties. BD: building direction. Reprinted from Andreiev et al. [87], with permission from Elsevier^®^.

In the study conducted by Andreiev et al. [87], they worked with a Fe–3.0%Si alloy processed by laser beam melting (LBM), aiming to evaluate a graded cross-section generated by incorporating grooves at various positions within the cross-section. Figure 3 shows the Computer-Aided Design (CAD) model of the samples, in which various slits were varied. In Figure 3a, the proposed five slits with sizes ranging from 50 μm to 250 μm are observed, while Figure 3b displays the specimen with a single slit of 50 μm, 100 μm, or 150 μm. Figure 3c illustrates the slit design on the ring used for assessing magnetic behavior for four different transverse sections. They found that the groove geometry can be controlled by adjusting the initial groove thickness defined in the CAD model, which should be greater than 150 μm. This control is further influenced by specific parameters within the lower layer (laser power−70 W and 100 W, along with a minimum requirement of eight processed layers or more). These conditions lead to the formation of grooves under conditions of porous structures or continuous gaps filled with unfused powder (approximately 390 μm). Additionally, a heat treatment of annealing at 550 °C for 2 h followed by air cooling was necessary to prevent bending of the walls between the outer grooves. The geometry and position of the grooves, whether on the internal or external surface or in a displaced manner, influence the power losses. This can be observed in the results, where grooves as continuous gaps on the external surfaces reduce the power losses from 19.7 W/kg to 11.2 W/kg at 50 Hz. However, the authors suggest that further reducing power losses below the values of conventionally laminated magnetic cores could entail several strategies for magnetic cores produced via this AM technique. These strategies include generating thinner grooves as nearly continuous gaps, using finer metallic powder (20 μm), increasing the silicon content in the alloy, and/or adapting the post-processing sequence.

Quercio et al. [88] fabricated Fe–2.9%Si samples using Laser Powder Bed Fusion (L-PBF) to evaluate the coercivity and permeability before and after annealing. For the coercivity test, rectangular and cylindrical bar-shaped components were fabricated. The results for the cylindrical shape showed a low coercivity value (69.1 A/m) even before treatment (87.7 A/m) compared to those obtained in the rectangular bar (212 A/m before treatment and 85.6 A/m after treatment). This behavior could be related to the geometric shape and/or printing conditions of a small diameter. The evaluation of relative permeability before heat treatment showed values of 748 and magnetic induction not exceeding 1.1 T. After heat treatment, the relative permeability increased to 3224, and the magnetic induction exhibited values higher than 1.2 T. These results indicate that the sample geometry and heat treatment directly influence the magnetic characteristics of the produced parts.

**Figure 4:**
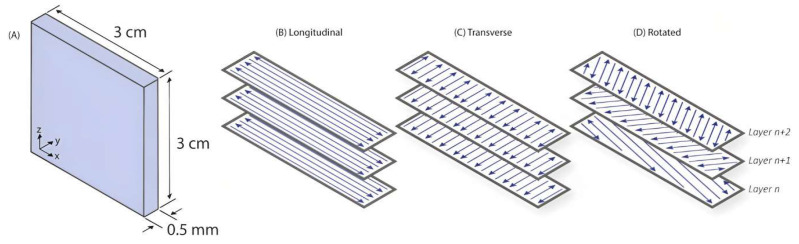
(**A**) Schematic illustration of the prismatic shape used for L-PBF processing; scan directions with reference to the top surface of the prism: (**B**) longitudinal; (**C**) transverse; and (**D**) rotated. Reprinted from Haines et al. [90], with permission from Elsevier^®^.

**Figure 5:**
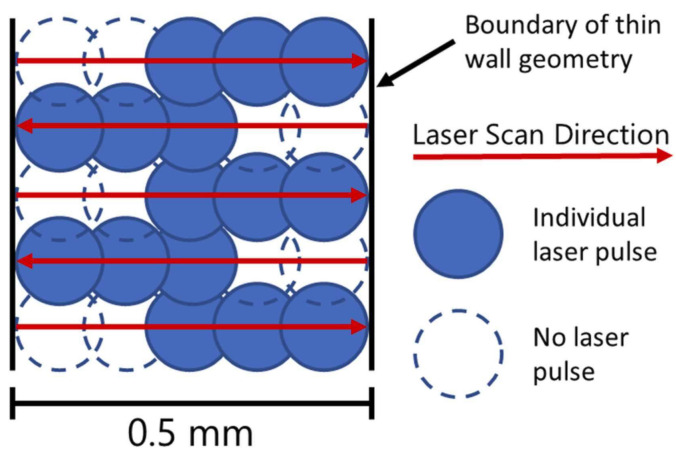
Schematic illustration of “point-skipping” during laser scanning. Reprinted from Haines et al. [90], with permission from Elsevier^®^.

Selema et al. [89] investigated the manufacturing of Fe–3%Si samples using two different AM techniques: 3D micro-extrusion and L-PBF. The results suggest that 3D micro-extrusion, coupled with a subsequent sintering process, allows the production of magnetic materials with improved properties, approaching those of conventional electrical steel. The study highlights the potential of AM techniques to open new frontiers for the electromagnetic sector. Regarding the experimentation carried out for the same Fe–3%Si via L-PBF technology, different scanning strategies were used for producing thin-walled samples by Haines et al. [90]. The main purpose was to examine how the scanning strategy would impact the microstructure of the alloy after undergoing an annealing heat treatment (argon atmosphere at 1000 °C for 5, 60, and 240 min and 1200 °C for 5 and 60 min using a 10 °C/min ramp rate and furnace cooling). Figure 4 illustrates the specimen sizes (Figure 4A) and the three distinct scanning strategies proposed by the authors. These include longitudinal scanning along the length of the part (Figure 4B), transverse scanning across the width (Figure 4C), and a rotated approach with a 67° rotation in the scanning direction between each layer (Figure 4D). A prominent aspect of the execution of each scan, called “point skipping,” is presented in Figure 5. This technique involves skipping two points every time the laser reverses during a scanning sequence. This technique helps reduce overheating in the area that has recently received an input of energy, resulting in improved process control and enhanced material properties. A comparison between the two scanning strategies revealed distinct differences in grain morphology and size. Specifically, the longitudinal scanning strategy leads to the formation of more equiaxed and/or misoriented grains with smaller dimensions than the transverse scanning strategy. On the other hand, the effect of annealing at different temperatures revealed distinct changes: at 1000 °C, the longitudinal samples experienced grain growth after 60 min, whereas at 1200 °C, grain growth occurred after 5 min. Additionally, the transverse scanning samples exhibited grain growth after 60 min. These findings highlight the temperature-dependent behavior of the material and the influence of the annealing and scanning strategies on its microstructure, indicating the importance of controlling the annealing parameters for achieving desired grain characteristics and optimizing material properties.

Martin et al. [91] conducted a study focused on the fabrication of Fe–3%Si toroidal cores using an additive manufacturing technique like MIM-like (Metal Injection Molding), which offers a more cost-effective approach. The aim was to demonstrate the feasibility of producing dense metal parts in Fe–3%Si through this process. The results indicated a relatively uniform surface on the sintered sample, without any significant defects or open porosities that could typically occur at high temperatures. Therefore, using this technique, it is now possible to effectively solve specific applications that previously faced challenges in terms of material manufacturability or geometric complexity.

**Figure 6:**
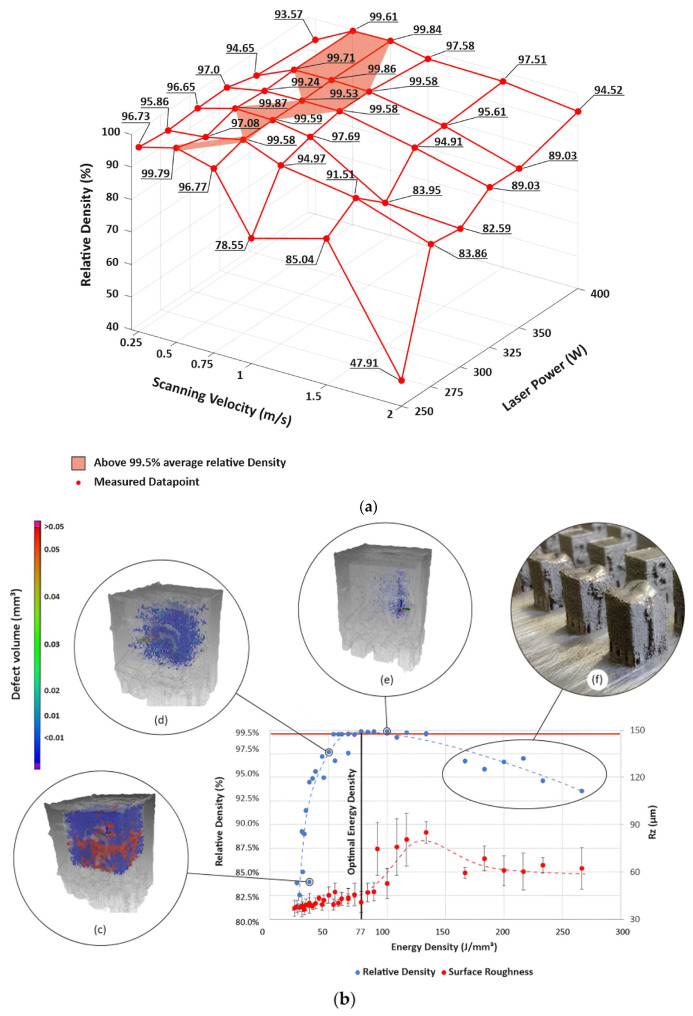
(**a**) Relationship between scanning parameters and relative density; (**b**) relative density and surface roughness as functions of laser input energy density; (**c**) sample at 300 W, 1.5 m/s; (**d**) sample at 300 W, 1 m/s; (**e**) sample at 300 W, 0.5 m/s; (**f**) samples at 400 W, 0.25 m/s. Reprinted from Tiismus et al. [137].

The works carried out by Tiismus et al. [92,137] present a comprehensive investigation of additive manufacturing using L-PBF to produce Fe–3.7%Si magnetic cores and subsequent applications. The study presented in [137] describes the adjustment of process parameters to achieve samples with appropriate density, surface roughness, and magnetic properties. Figure 6 provides an overview of the correlation between scanning parameters, with a focus on laser input energy, and the resulting relative density and surface roughness of the manufactured samples. In Figure 6a, it can be observed that the lowest relative density value (47.91%) is achieved at 250 W and 2 m/s, while at 300 W and 0.5 m/s (78 J/mm^3^), a notably high relative density of 99.86% is attained. Figure 6b suggests that the average surface roughness of the samples (ranging from 6.8 to 18.2 µm for Ra and 36.1 to 85.2 µm for Rz) does not exhibit a monotonic increase with rising laser input energy density. Moreover, Figure 6c demonstrates that within the 20–50 J/mm^3^ range, the energy supplied under specific parameter sets is insufficient to achieve uniform fusion (300 W, 1.5 m/s). As scanning speed is decreased, homogeneous fusion of samples is achieved (Figure 6d,e). Notably, it is important to highlight that with increased laser power (250–400 W, 0.25 m/s), samples experience deformation due to the heightened energy input (Figure 6f). XRD results indicate the formation of a single α-ferrite BCC phase, and the formation of the γ phase is suppressed with increasing silicon content during solidification. Finally, the authors conducted direct current measurements, obtaining hysteresis losses of 0.8 W/kg (W10,50), a maximum relative permeability of 8400, and magnetizations of 1.5 T at 1480 A/m and 1 T at 90 A/m. These results were correlated with values in the magnetic properties of conventional steels, showing equivalency to the latter. The application of these results can be visualized in further research [92], which focused on the development of an induction motor with L-PBF printed Fe–3.7%Si cores. The performance evaluation of the finished motor showed an output power of 68 W from the steel core. To address the challenge of minimizing eddy current losses, an innovative segregation strategy was implemented by creating a uniform and narrow air gap of 0.35 mm perpendicular to the core axes.

Additionally, lattice structures were added in these gaps to consolidate the printed part into a single core, preventing deformation and delamination during printing and promoting powder fusion between the layers. A thorough understanding of these effects is crucial for optimizing L-PBF process parameters and achieving the desired microstructural features and performance in 3D-printed Fe–Si alloys.

It is essential to mention the influence of chemical composition and microstructural changes on the magnetic properties of components manufactured through additive manufacturing. In a study conducted by Kumary et al. [93], the impact of Si and B content on the properties of Fe–Si–B alloys fabricated through binder jet printing was investigated. The Si content ranged from 3 to 5 wt.%, while the B content ranged from 0.0 to 0.25 wt.%. The Fe–5.0%Si alloy with 0.25% B, sintered at 1200 °C, demonstrated the highest magnetic relative permeability and the lowest intrinsic coercivity. The addition of B led to larger grain sizes with reduced porosity and increased density, attributed to the formation of the ferromagnetic lamellar phase Fe_2_B at the grain boundaries.

Kang et al. [94] undertook a study on Fe–Ni-Si alloys with compositions of 54%, 42%, and 4% in weight proportions using L-PBF technology. The study explored the impact of the hot isostatic pressing (HIPing) process on a reduction in porosity, with a focus on eliminating small pores while larger pores remained even after the HIPing process. During HIPing, the acicular Ni/Si-rich structure, which forms in the L-PBF fabricated samples due to the rapid cooling rates, undergoes a transformation of equilibrium phases such as austenite, Ni_3_Si, and FeNi_3_. The study also assessed the mechanical properties of the samples, showing an increase in both the elastic modulus and strength, from about 11 GPa and 650 MPa up to 18 GPa and 900 MPa after HIPing. Furthermore, the fitted data indicated an increment in coercivity from approximately 1.8 kA/m up to 2.9 kA/m.

At the beginning of this section, a general overview is provided regarding the advantages and limitations of these alloys when increasing the silicon content. It is considered high-silicon steel when the percentage of this element is ≥4.5 wt.% [139,140]. This increase in Si provides the steel with remarkable magnetic properties such as high electrical resistivity, reduced magnetostriction, and magneto-crystalline anisotropy, making them ideal for the mentioned components [39,95,141,142]. The main limitation of high silicon steels lies in their brittleness when forming phases with ordered structure (B2 and D03) during the cooling stage of manufacturing, which reduces the ductility of the material and limits its machinability using traditional forming processes. These phases represent two types of ordered structures commonly formed in high-silicon-content steels. The B2 structure arises from the A2 (a disordered solid solution with a BCC lattice) by pairing different atoms from the nearest neighbors. With an increase in silicon content in the alloy, a phase transition towards the ordered D03 structure might occur through further arrangement among second-order neighboring atoms [42,143]. The following section will address various works on these materials using AM. We focus on the influence of process parameters, design or orientation of printed geometries, and heat treatment, among others, on changes in crystallographic texture, microstructure, hardness, and magnetic properties. These research studies demonstrate the significant impact of additive manufacturing in the processing of these materials.

**Figure 7:**
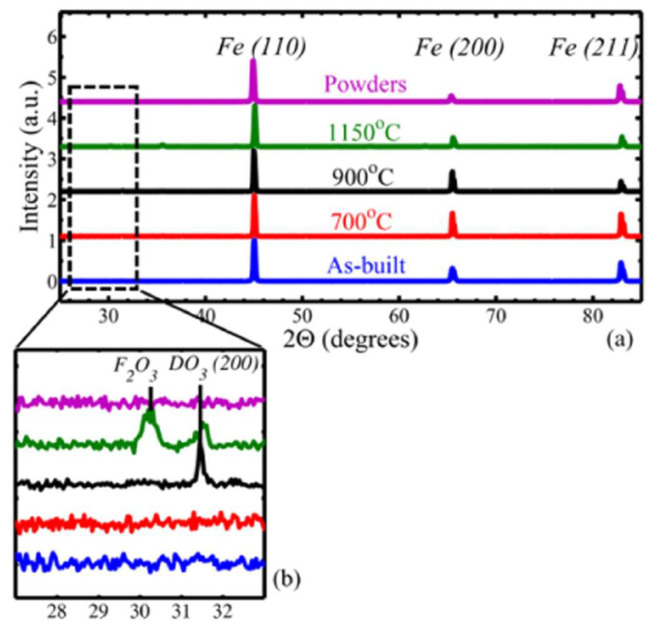
XRD before and after annealing at various temperatures. (**a**) complete XRD spectra; (**b**) amplified extracts of the spectra. Reprinted from Garibaldi et al. [97], with permission from Elsevier^®^.

The studies conducted by Garibaldi et al. [96,97] are a reference point for addressing this topic. In one of their research projects [96], they specifically investigated the effect of laser energy in the selective laser melting (SLM) process on the magnetic properties of high-silicon-content steel samples (6.9%). An interesting starting point in this study was the choice of the manufacturing orientation of the components. By constructing the samples with their bases parallel to the horizontal plane, it was possible to measure the magnetic properties of the material in its isotropy plane. The SLM process promotes the ⟨001⟩ crystallographic direction (easy magnetization axis), resulting in isotropic magnetic properties within the horizontal plane of an SLM sample. In contrast, the ⟨111⟩ direction (the hard direction of magnetization) tends to be outside the horizontal plane. Using the Williamson–Hall method, it was determined that annealing the samples at 700 °C for 5 h allowed for the complete elimination of residual stresses. However, Vickers microhardness (HV) analysis revealed a significant increase in HV after this treatment. This increase can be attributed to the redistribution of silicon-rich microsegregations and the relief of internal stresses. The results of the magnetic characterization showed that it was possible to achieve a maximum relative permeability of 5300, a coercivity of 49 A/m, and power losses of 4 W/kg using a laser power of 70 W and a scanning speed of 0.250 m/s. The authors provided a detailed explanation of these results based on the crystallographic texture in [95]. According to these studies, processing Fe–6.9%Si using SLM with low laser energies results in a fibrous ⟨001⟩ texture. As the laser power increases, the texture changes to a cubic texture due to epitaxial grain growth and a shift in the shape of the melt pool from a surface depression to a protrusion. As the texture becomes cubic, the easy magnetization axis aligns with the direction of the magnetic field only at approximately 45° to the laser scanning directions. This causes the horizontal plane to lose its isotropic nature, leading to an increase in hysteresis loss, a decrease in remanence (B_r_), and a decrease in permeability as the alignment between the magnetic field and the ⟨001⟩ axes becomes less pronounced. Through the other investigation on Fe–6.9%Si samples processed using SLM [97], they examined the effect of annealing parameters on the microstructural and magnetic development of the components. They employed a laser power of 70 W, a laser scanning speed of 500 mm/s, and four different annealing temperatures (400 °C, 700 °C, 900 °C, and 1150 °C) in an argon atmosphere for 1 h, followed by cooling in the furnace. The microstructure of the samples prior to annealing indicated a grain size of 10–30 μm, which remained unchanged after annealing at 700 °C. However, after annealing at 900 °C, several significant aspects emerged in the investigation of these materials: (1) A partially recrystallized microstructure formed due to the release of residual stresses. (2) At this annealing temperature, the dissolution of Si led to the complete disappearance of fusion pool boundaries. Finally, the microstructure at 1150 °C exhibited a non-uniform grain size, with large equiaxed grains (up to 300 μm) and smaller grains (<30 μm). The authors suggested that the recrystallization process was incomplete. Figure 7 shows the XRD analysis conducted in this study, revealing a single BCC phase before and after annealing as well as a superlattice diffraction line related to the D03 phase ordering in the samples annealed at 900 °C and 1150 °C.

The authors concluded that the high cooling rates experienced during SLM partially suppressed diffusion-driven ordering reactions based on the Fe–Si binary alloy phase diagram. The D03/B2 ordered phases in Fe–6.9wt% Si are thermodynamically stable below 830 °C. On the other hand, EBSD analysis indicated that annealing did not significantly alter the ⟨001⟩ texture generated by SLM along the build direction. The analysis of annealing effects and their relationship with magnetic properties through microstructural changes revealed that the maximum relative permeability increased (from 2000 to 24,000) with the increase in annealing temperature. This was associated with stress relief and grain growth effects. The coercivity values were observed at 16 A/m, achieved by increasing the annealing temperature, while the residual M_r_ decreased as the temperature increased.

This increase also leads to a variation in the maximum flux density (B_MAX_). Furthermore, the microstructural analysis shows that the average grain size tends to increase slightly with the increase in E_0_*, while the coercivity (H_C_) decreases as the average grain size increases. The evaluation of texture to assess the correlation between the average magnetocrystalline energy and the magnetic properties (H_C_, B_MAX_) demonstrates a weak effect of texture, which can be attributed to the high level of magnetization. This indicates that, in this study, the crystallographic texture has a secondary effect compared to density and grain size.

**Figure 8:**
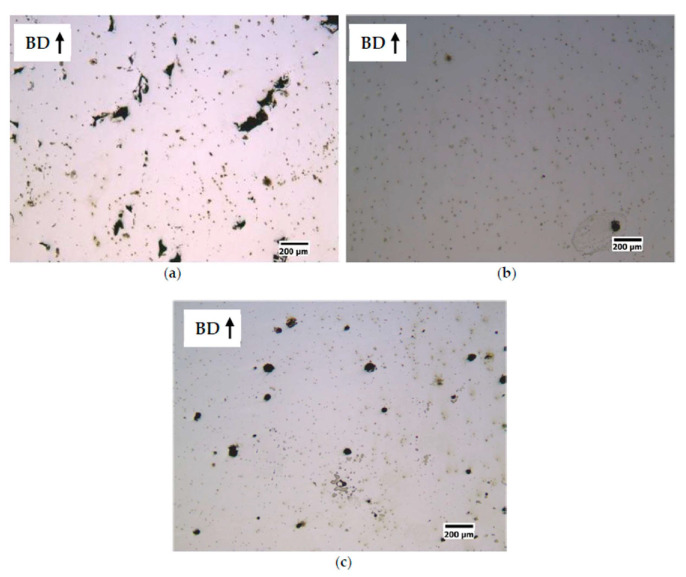
Effect of the specific laser energy (E) on the densification of Fe–3.0%Si steel samples. (**a**) E = 150 J/m, v = 0.5 m/s, P = 75 W, relative density 99.93%, and pores with irregular shape; (**b**) E = 250 J/m, v = 1 m/s, P = 250 W, relative density 99.99%; (**c**) E = 350 J/m, v = 0.5 m/s, P = 175 W, relative density 99.98%, and pores with spherical shape. Reprinted from Stornelli et al. [101].

**Figure 9:**
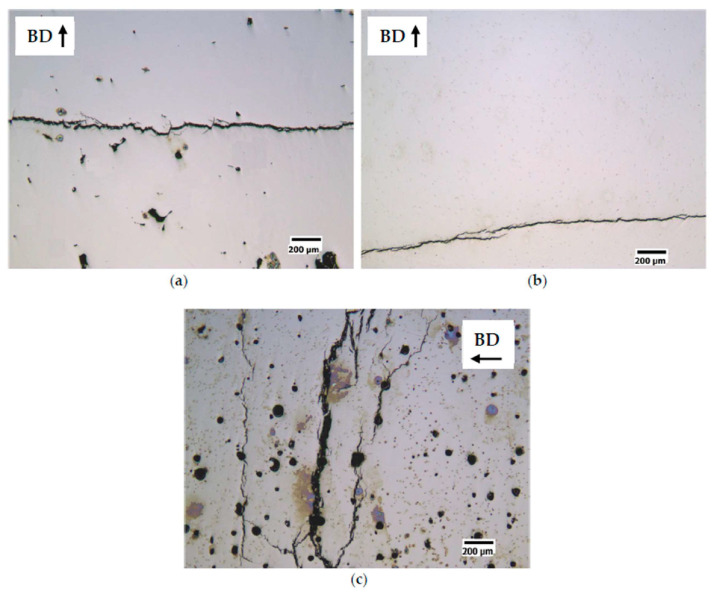
Effect of the specific laser energy (E) on the densification of Fe–6.5%Si samples. (**a**) E = 150 J/m, v = 0.5 m/s, P = 75 W, relative density = 99.93%, and pores with irregular shape; (**b**) E = 200 J/m, v = 0.835 m/s, P = 167 W, relative density = 99.99%; (**c**) E = 350 J/m, v = 0.5 m/s, P = 175 W, relative density = 99.98%, and pores with spherical shape. Reprinted from Stornelli et al. [101].

The works of Stornelli et al. [99,100,101,102] in these types of alloys, through microstructural and magnetic studies, have provided results of relevant importance. In [101], they conducted a study to identify the optimal process parameters for L-PBF of Fe–Si steels with Si contents of 3.0% and 6.5%, aiming to evaluate their effect on the density, microstructure, and electromagnetic properties of the printed samples. The authors found that to reduce the internal porosity fraction in the Fe–3.0%Si steel, the suitable process parameters were E = 250 J/m, v = 1 m/s, and P = 250 W (Figure 8b). The reported values for the Fe–6.5% steel were E = 200 J/m, v = 0.835 m/s, and P = 167 W (Figure 9b). In terms of relative density values, both steels exhibited values close to 1. Microstructural results showed that the printed samples maintained a columnar solidification microstructure aligned with the build direction due to the epitaxial growth of the previously consolidated material in the underlying layers. The influence of the process parameters can be observed based on the laser-specific energy (E), where low energy favors the formation of irregular-shaped pores (Figure 8a and Figure 9a). This shape can be associated with voids between partially melted powder particles. On the other hand, high energy levels lead to spherical-shaped pores resulting from the capture of metal vapor and partially ionized gas in the liquid pool, which solidify into a spherical cavity upon rapid cooling. It is important to mention that crack formation was not observed in the Fe–3.0%Si steel. At the same time, an increase in E resulted in more noticeable crack formation in the Fe-6.5% samples (Figure 9c), which could be attributed to the high thermal gradients. As a future work, the authors suggest raising the platform temperature to reduce thermal stresses generated by the process’s nature. Magnetic analysis was performed on different cross-sectional geometries of samples from both steels. This analysis revealed that the Fe-6.5% samples exhibited lower effects of eddy currents compared to the Fe–3.0%Si samples, resulting in an increase in magnetization capacity and a more than 50% reduction in power losses. The results obtained in this research demonstrate the ability to produce ferromagnetic cores with high Si content and attractive electromagnetic properties through AM, offering a competitive alternative for industrial applications.

Seiler et al. [103] investigated the influence of high-frequency modulation through the combination of continuous laser power (high build speed) and pulsed laser power (reduction in input energy) in the L-PBF process for Fe–6.7%Si samples to evaluate its effect on the surface topography of the samples. The results of this research show incomplete fusion of the powder layers (with a thickness of 20 µm) when using laser power below 75 W. However, although increasing laser energy promotes the complete fusion of powder particles in the sample and the attainment of a nearly fully dense structure, temperature gradients and the increase in residual stresses generated by higher laser power contribute to the formation of cracks in the studied material.

An investigation made by Agapovichev et al. [104] through the use of the L-PBF process to produce the samples from Fe–6.5%Si powder shows that even at the best optimization of the process parameters, cracks may appear. In fact, carbon content was detected at the boundaries of the cracks. This suggests that one of the reasons for the crack formation is the presence of Fe_3_C around the ordered αFe–Si (B2) + Fe_3_Si(D03) phases.

Goll et al. [105] have conducted research on the processing of high-silicon steels using L-PBF for soft magnetic core materials such as Fe–6.7%Si. The objective was to reduce eddy current losses and achieve good magnetic performance. In this work, a laser power of 300 W and a laser scanning speed of 500 mm/s were used to reduce the number of pores, increase the grain size, and prevent crack formation. The research results indicate that a larger grain size can be obtained by using higher laser power due to the decrease in cooling rate. However, special attention must be given to avoiding/reducing crack formation, which becomes more pronounced at higher power levels due to increased residual stresses. Along with the mentioned parameters, maintaining the platform heating temperature at 400 °C allows for high electrical resistivity (0.8 μΩm), maximum permeability (31,000), minimum coercivity (16 A/m), and hysteresis losses (0.7 W/kg at 1 T and 50 Hz). Furthermore, a significant aspect of the study was focused on evaluating the design flexibility offered by AM, specifically in the context of component design. The researchers explored the utilization of topological structures, such as inner slits, to take advantage of AM’s capability to create complex geometries. These topological structures were strategically incorporated into the components to interrupt the paths of electrical current, reducing eddy currents and minimizing the adverse effects of Foucault currents. This aspect of the study highlights the inherent advantage of AM, where the freedom in design allows for the incorporation of optimized structures that are challenging to achieve through traditional manufacturing methods.

The results from various investigations presented in this section provide insight into the scope of additive manufacturing in Fe–Si alloys. Different processes such as L-PBF, LBM, SLM, DED, and even innovative methods like MIM have been employed to fabricate components based on this alloy, encompassing various geometries. This showcases AM’s capacity to produce components with intricate geometries. The impact of process parameters, such as scanning orientation, laser power, scanning speed, or their correlation through laser energy input, is observed in the resultant microstructure. This microstructure typically consists of columnar and/or equiaxed grains, with the possible presence of discontinuities like pores, cracks, or a lack of fusion. These aspects are linked to gas entrapment, excessive energy input, or insufficient energy for the complete fusing of the manufactured piece. Moreover, microstructural analyses were conducted using characterization techniques such as SEM, EBSD, or XRD to assess the distribution of chemical elements based on process parameters, crystalline orientation, and the presence of ordered B2/D03 phases that could affect the alloy’s mechanical behavior. The evaluation of magnetic properties, including parameters like magnetic saturation and coercivity, reveals competitive outcomes when compared with values from Fe–Si alloys processed using traditional techniques. Notably, additive manufacturing demonstrates the capability to process alloys with high silicon content, allowing the creation of various geometries with remarkable magnetic and mechanical properties. Even though there are flaws in some of the results, there are still areas for improvement. For instance, post-processing conditions could be improved or fusion bed manipulation could be modified to achieve defect-free components or reduce their occurrence.

## 4. Fe–Ni

The iron–nickel alloys (Fe–Ni) can be classified based on their nickel content. Among them, “permalloys” stand out for their excellent soft magnetic properties, including low power losses, high permeability, and a favorable response to magnetic field annealing. Although permalloys offer significant advantages for high-frequency electrical machines, their implementation in electric motors is less frequent due to their higher cost compared to Fe–Si steel. Other important Fe–Ni alloys are the “supermalloys,” characterized by their high nickel content of around 78%, 5.0% molybdenum, iron, and a small amount of manganese. They also possess exceptional magnetic properties, including superior permeability, low losses, and excellent response to magnetic field annealing. To optimize their permeability, precise heat treatment is required, involving specific temperatures and controlled cooling rates. Supermalloys may cost more than permalloys and silicon steels, but their exceptional properties make them a promising alternative for high-frequency electrical machines, where their superior performance justifies the investment [144,145,146,147,148].

**Figure 10:**
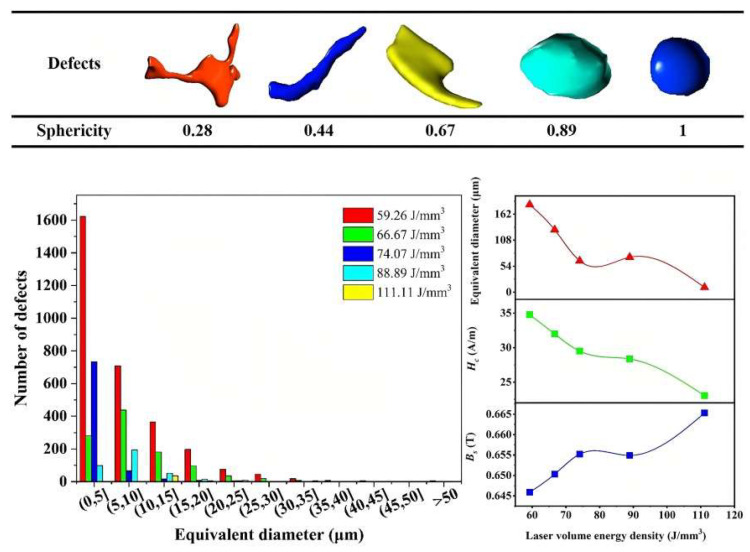
The sphericity of the typical defects, equivalent diameter distribution, and curves of H_c_ and B_s_ in the Ni–Fe–Mo samples fabricated at different laser volume energies. Reprinted from Yang et al. [112], with permission from Elsevier^®^.

Additive manufacturing techniques have been employed for the processing of Fe–Ni-based alloys [106,107,108,109,110,111]. The importance and notable characteristics of these alloys can be verified in various studies. Permalloy samples of Ni–Fe–Mo fabricated using SLM were produced with different process parameters to study the correlation between these parameters, the morphology of generated defects, and the magnetic properties, as can be observed in [112]. Laser power in the range of 160–240 W and scanning speeds of 600–1000 mm/s were utilized, with a hatch space of 0.1 mm and a layer thickness of 0.03 mm. This set of parameters was correlated through an equation to obtain input laser energy densities (E_V_), resulting in values ranging from 59.26 to 111.1 J/mm^3^. As E_V_ increases, there is an increase in magnetic saturation induction and magnetic permeability of the material, accompanied by a reduction in coercivity and magnetic losses. Figure 10 depicts the 3D reconstruction using X-ray computed tomography (XCT), an important aspect of this research.

**Figure 11:**
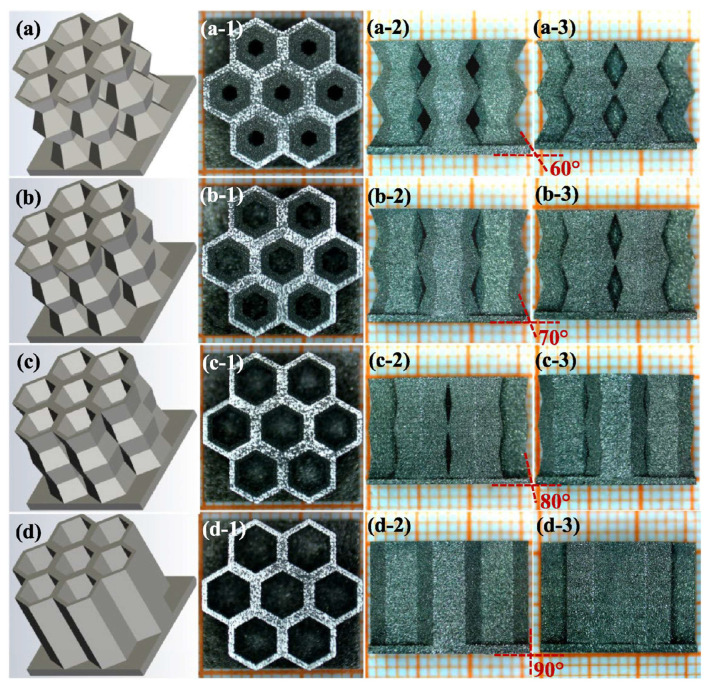
Macro-morphologies of modified honeycomb prototype parts with 0.5 mm walls and overhanging angles of 60° (**a**), 70° (**b**), 80° (**c**), and 90° (**d**) in 3D models (**a**–**d**), top views (**a-1**–**d-1**), front views (**a-2**–**d-2**), and side views (**a-3**–**d-3**). Reprinted from B. Li et al. [113], with permission from Elsevier^®^.

The authors illustrate sphericity (S), which is calculated through a formula that relates the volume and surface area of the defect that the sample presents and whose result will oscillate between values of 0 and 1 (where 1 denotes higher sphericity). The outcomes indicate that with a low E_V_ value (59.26 J/mm^3^), microdefects displayed irregular shapes due to incomplete fusion. In contrast, with high E_V_ (111 J/mm^3^), these microdefects exhibited circular forms attributed to gas-induced porosities. The distribution of the equivalent diameter (D_eq_) of defects in samples fabricated with varied E_V_ values is also shown, revealing a significant reduction in defect count as E_V_ increased. Finally, Figure 10 also presents the correlation between H_C_ and B_S_ with the D_eq_ of internal metallurgical defects at different E_V_ levels, demonstrating a clear decrease in H_C_ and an increase in B_S_ as the D_eq_ diminishes. The study by B. Li et al. [113] demonstrated the feasibility of producing modified honeycomb parts of Ni–15%Fe–5%Mo permalloy through L-PBF (Figure 11). The results highlighted the importance of optimizing process parameters and the unique microstructural features obtained, which could have significant implications for magnetic shielding applications. The as-printed permalloy bulk samples achieved a remarkable relative density exceeding 99.5%. The researchers successfully printed thin walls with varying thicknesses and different overhanging angles using L-PBF technology. Microscale or even submicron-scale cellular structures were observed within the grains, demonstrating growth through multiple molten pools. Notably, the modified honeycomb structures demonstrated preferential plastic deformation at the inclined corners. This characteristic allowed for better plastic deformation buffering effects and prevented instantaneous cracking.

The studies conducted by Kim et al. [114,115] extensively demonstrate the effect of additive manufacturing parameters on the properties of these soft magnetic alloys. In [114], they conducted an evaluation of Fe–50%Ni samples manufactured using direct energy deposition based on lasers under various process conditions. These conditions include laser power ranging from 200 to 220 W, a powder feeding rate of 2.25–2.75 g/min, a laser scan speed of 100–1000 mm/s, and hatch spacing of 0.06–0.3 mm. The microstructural analysis revealed that the samples exhibited a single FCC phase with epitaxial columnar grains. Increasing the laser power to 220 W induced a change in the solidification mode, resulting in larger grain sizes. At this laser power level, the sample demonstrated favorable mechanical and magnetic properties, including a hardness value of 167 HV, a maximum tensile strength of 493 MPa, a yield strength of 315 MPa, a saturation magnetization of 151 emu/g, and a minimum coercivity of 3.16 Oe. These properties make the Fe–50%Ni alloy competitive with other soft magnetic alloys in terms of performance. In another study [115], three energy densities (4000, 4800, and 5867 J/g) based on the process parameters from the previous study were used. Subsequently, the samples were heated to 1200 °C (the temperature at which only the FCC phase exists) for 1 h and cooled in a furnace. The results revealed that after annealing, the sample manufactured with an energy density of 4800 J/g maintained the FCC phase structure. In comparison, a secondary ferrite phase (BCC microstructure) and lower microhardness values were observed in the samples manufactured with energy densities of 4000 and 5867 J/g, respectively. This reduction in microhardness is associated with grain growth, partial stress relaxation, recovery of the cellular structure, and especially the formation of a softer BCC phase. It is noteworthy that the sample processed at 4800 J/g and annealed exhibited outstanding values of coercivity, saturation magnetization, and Curie temperature of approximately 1.8 Oe, 170 emu/g, and 530 °C, respectively. This is attributed to the unique microstructure of the FCC phase, lower concentration of grain boundaries, and lower deformation level after annealing.

**Figure 12:**
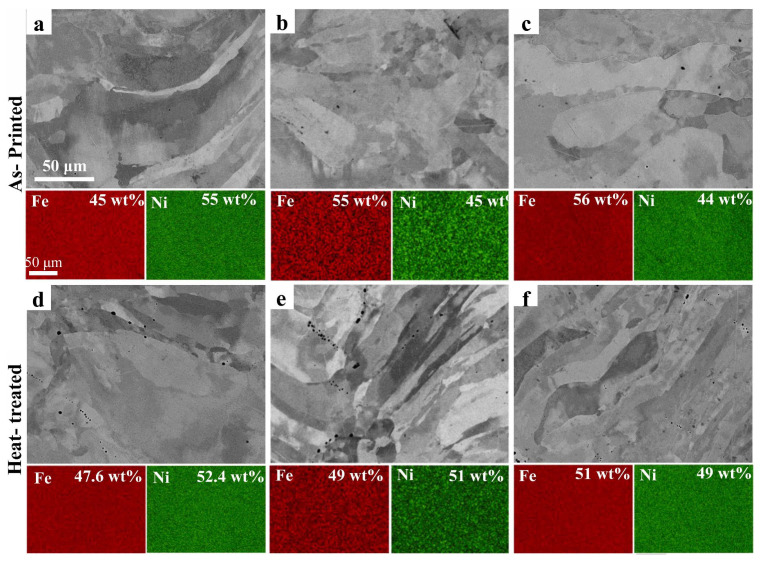
SEM images of DED samples with rotation: (**a**,**d**) 67°, (**b**,**e**) 90°, and (**c**,**f**) no rotation (black spots are metallic oxides trapped inside). Reprinted from Haftlang et al. [116], with permission from Elsevier^®^.

The crystallographic texture plays a crucial role in the magnetic behavior of materials, particularly in the case of soft magnetic materials processed through additive manufacturing. Post-processing treatments, such as heat treatments and orientation changes during component fabrication, offer a wide range of possibilities to control and optimize the crystallographic texture. This was demonstrated in the study conducted by Haftlang et al. [116] on the crystallographic texture in Fe–50%Ni permalloy samples using the DED process. To evaluate this topic, they employed two rotation strategies (67° and 90°) and processed the sample without rotation (NR). Additionally, after printing, the specimens underwent a heat treatment at 1200 °C for 60 min in a pure Ar atmosphere. The process parameters included laser power ranging from 200 to 220 W, powder feeding rate from 2.2 to 2.75 g/min, and laser scan speed from 100 to 1000 mm/s. Figure 12 presents SEM images and the results of Energy-Dispersive X-ray Spectroscopy (EDS) analysis conducted on the manufactured samples. The outcomes reveal a fluctuation in the Fe percentage contingent on the processing rotation (67° = 45 wt.% Fe, 90° = 55 wt.% Fe, and NR = 55 wt.% Fe), which can be attributed to Fe/Ni micro-segregation at the fusion pool boundaries. Moreover, Figure 12 demonstrates that subjecting the samples to subsequent heat treatment results in a uniform chemical composition, irrespective of the processing rotation. The effect of rotation was also observed in the microstructural evolution of the parts. Without rotation, a heterogeneous microstructure with fine, inclined columnar grains was obtained. In contrast, rotations of 67° and 90° resulted in grain sizes of 70 µm and 93 µm, respectively, with elongated and wavy structures due to the variation in heat flow direction. The results of the texture analysis conducted by the authors yielded important findings. The sample without rotation exhibited predominant Cube {001} ⟨100⟩ and Copper {112} ⟨111⟩ texture components. With a rotation of 67°, the sample shows a pronounced γ_γ_-fiber ({111} ⟨uvw⟩) component at φ_2_ = 45° and a pronounced S_γ_ ({123} ⟨634⟩) component at φ_2_ = 65°. In contrast, the sample with a 90° rotation exhibited predominant Cube {001} ⟨100⟩ and Brass {011} ⟨211⟩ texture components. It is important to mention that, based on their results, the authors indicated that the heat treatment did not significantly change the grain morphology of the printed samples. However, it did increase the intensities of the main texture components, while the intensities of non-ideal orientations decreased. Furthermore, its effect was manifested in its influence on magnetic properties, as the sample without rotation exhibited values of Hc = 1.7 Oe, Ms = 160 emu/g, and Tc = 540 °C.

Mohamed et al. [117] evaluated the combined impact of subsequent heat treatment and control of crystallographic texture in a Ni–Fe–Mo alloy processed via L-PBF. They generated cubic samples that were tilted along the [103] and [104] directions with respect to the building direction, corresponding to 45° and 35°, respectively. This was achieved using a range of parameters that included laser power from 150 to 300 W, scan speed from 800 to 3500 mm/s, and scan spacing from 0.2 to 0.6 mm. Consequently, these parameters contributed to an energy density spectrum ranging from 0.95 to 6.25 J/mm^2^. Four types of heat treatments were performed to evaluate their effect on different batches of parts. The first treatment (HT) involved heating to 1150 °C for 4 h at a rate of 2 °C/min in a hydrogen atmosphere. When it reached 550 °C, it was held for 1 h, then increased to 1150 °C and held for 4 h before being reduced to 200 °C at a rate of 0.8 °C/min. The second treatment, hot isostatic pressing (HIP), was carried out at 1230 °C and 120 MPa for 3 h (with simultaneous application of pressure and temperature) at a rate of 5 °C/min. The third and fourth treatments combined the first two, with only the sequence being varied: (3) HT + HIP and (4) HIP + HT. The results showed that the inclination of the build orientation promoted an improvement in the normal and transverse magnetic shielding characteristics in the main build directions. The samples exhibited recrystallization and grain growth, transitioning from columnar grains extending for 10 µm along the build direction to equiaxial grains after the application of heat treatments. Moreover, HT, HIP, and their combinations resulted in stress relief, a reduction in inclusions, and pore consolidation, directly influencing the magnetic properties of the samples in all build directions. This can be seen in the results of M_S_ and H_C_ for the samples subjected to HIP + HT. For example, specimens printed with a 35° orientation exhibited attractive values of M_S_ = 637 and H_C_ = 195, compared to values of M_S_ = 542 and H_C_ = 205 for 45° and M_S_ = 561 and H_C_ = 207 for 0° orientation samples.

As previously mentioned, Fe–Ni alloys stand out as SMM with suitable magnetic characteristics for applications requiring high frequency. The findings from the investigations explored in this section underscore the necessity of post-manufacturing heat treatment for Fe–Ni alloys involving conventional annealing, processes like hot isostatic pressing (HIP), or their combinations. The impact of printing orientation, laser power, and scanning speed emerges prominently in both magnetic behavior and microstructural development. Several research studies have indicated that as energy input increases, magnetic saturation and permeability increase while coercivity decreases. Furthermore, the analysis of crystallographic texture has revealed its correlation with printing orientation. This allows the identification of planes where the alloy’s magnetic performance could achieve outstanding values. A vital element in analyzing these materials is defect reconstruction through XCT (X-ray computed tomography), as this enables the visualization and establishment of the relationship between the obtained discontinuities and the utilized parameters.

## 5. Fe–Co

Iron cobalt alloys (Fe–Co) possess excellent characteristics such as high saturation magnetization, high permeability, low magnetic losses, and a high Curie temperature, making them valuable in applications that prioritize thermal stability. It is important to highlight the highest magnetic saturation value for this alloy (approximately 2.4 T), surpassing other soft magnetic iron alloys. However, the conventional production of Fe–Co iron cores faces challenges such as the high cost of cobalt, the limited workability of the iron–cobalt alloy, and the necessity for heat treatment during manufacturing [55,111]. The mechanical strength, iron losses, and magnetic permeability of Fe–Co alloys can be controlled through various means. One approach is to modify the ratio of cobalt and iron as well as add other alloying elements. Another method involves modifying the temperature cycle during the annealing heat treatment. These operations allow for the obtaining of Fe–Co alloys in different forms, such as “Permendur,” “Supermendur,” and “Hiperco,” with compositions very similar to each other [118,119,120,121]. By exploring innovative approaches and techniques, such as AM, it is possible to overcome these issues, open new possibilities for optimizing the manufacturing process, and exploit the full potential of Fe–Co alloys in various applications.

The work presented by Lindroos et al. [122] aims to investigate the effect of process parameters in Laser Powder Bed Fusion (L-PBF) and post-manufacturing heat treatment on Fe–Co binary and ternary alloys (Fe–35%Co, Fe–50%Co, Fe–49%Co–2%V, and Fe–49%Co–2%V–0.1%Nb). The study also focuses on the development of mesostructures to mitigate eddy currents. This paper mentions the use of a laser power of 200 W, a scanning speed of 775 mm/s, and a layer thickness of 80 µm, which resulted in a reduction in porosity to below 0.1% in all cases. The effect of heat treatment varies for each material. For the Fe–Co–V ternary alloys, an optimized two-stage heat treatment (pre-annealing at 700 °C for 2 h followed by annealing at 820 °C for 10 h) generates a structure with uniformly large grain sizes. In contrast, conventional heat treatment (annealing at 820 °C for 4 h) leads to bimodal grain growth. It is noteworthy that the size of the printed samples plays a crucial role in the final microstructural and magnetic characteristics after annealing, as it affects the differences in the thermal history generated by the L-PBF process. For example, in large samples (20 × 20 × 65 mm), the microstructure consists of approximately 90% large grains, compared to 65% large grains in smaller samples (10 × 10 × 35 mm). Additionally, the specimen with a higher percentage of large grains achieves high magnetic induction, while the sample with fewer large grains exhibits low permeability. However, varying the annealing holding time (24 h) shows that it is possible to obtain almost identical microstructures in both sample sizes, leading to good magnetic performance. The addition of Nb to the Fe–Co–V alloy helps reduce grain growth. The addition of 0.1% Nb to the L-PBF printed samples results in a 50% smaller grain size compared to samples processed without this element. On the other hand, in the Fe–35%Co and Fe–50%Co binary alloys, the effect of different heat treatments is less significant, and the grain size remains almost unchanged when using either conventional or optimized heat treatment. The manuscript discusses the study of two mesoscale structure designs to limit eddy currents: (1) Slotted structures and (2) Topological optimization (TO) in defining the optimal cross-sectional shape for magnetic flux. The effect of these designs is exemplified by the coercivity values of Fe–Co–V alloys, which are 118 A/m for slotted structures and 74 A/m for TO, while the permeability values are 5148 and 3489, respectively. The recent study by Riipen et al. [123] investigated the influence of heat treatment and Nb addition on the chemical composition of a Fe–Co–V alloy processed by L-PBF. The authors used a laser power of 200 W, a scan speed of 775 mm/s, a hatch spacing of 80 µm, and a build platform temperature of 200 °C. The annealing heat treatment consisted of heating at 800 °C and 850 °C for 1 h, 10 h, and 24 h, followed by furnace cooling (cooling rate of 100 °C/h). The results of the microstructural evaluation show an irregular, small grain structure in the untreated samples. In contrast, the annealed samples show a bimodal grain structure even after 24 h, but it is noticeable that the proportion of small grains decreases with increasing annealing temperature and time. Furthermore, the influence of Nb can be observed in grain growth. In the sample with Nb addition, annealed at 800 °C and 850 °C after 24 h, the average grain size was smaller (45 µm) than in the sample without Nb (210 µm) at the same annealing temperature, but whose growth stopped after 10 h. Based on these results and investigations, the authors indicate the ability of Nb to inhibit grain growth. This effect is achieved by binding the particles at the grain boundaries through the precipitation of nano-sized particles in this zone. This precipitation is induced by a slight difference in chemical composition. The evaluation of the magnetic properties shows that heat treatment at 800 °C for 24 h gives optimum values of relative permeability (15,000) and coercivity (20 A/m) for the alloy with Nb addition. In contrast, for the alloy without Nb, these values are obtained at a higher annealing temperature (850 °C for 24 h).

**Figure 13:**
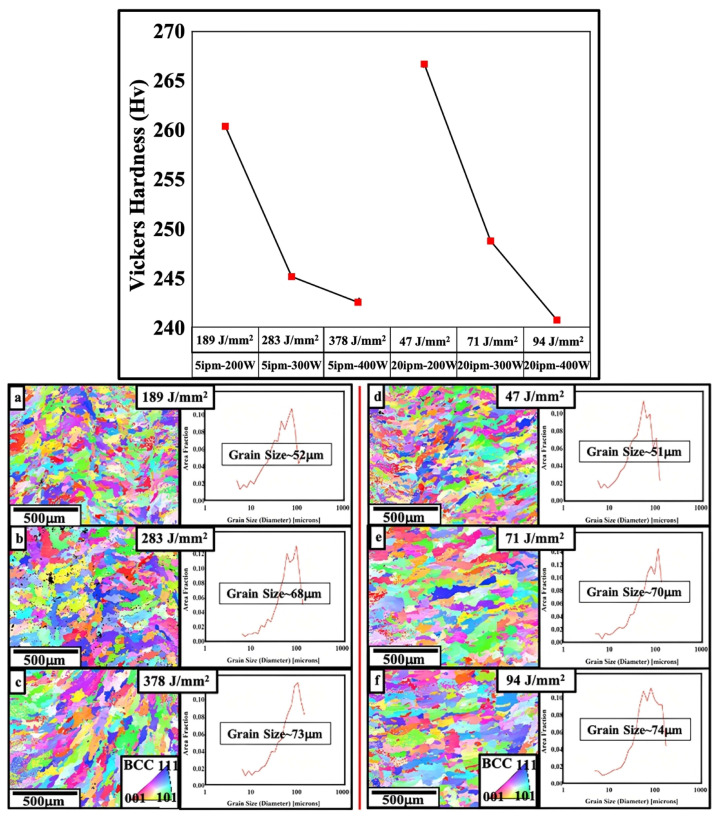
Vickers microhardness measurements for all six as-processed samples in Fe–Co–2V alloy, Inverse Pole Figure (IPF), and grain size distribution maps for (**a**) 189 J/mm^2^, (**b**) 283 J/mm^2^, (**c**) 378 J/mm^2^, (**d**) 47 J/mm^2^, (**e**) 71 J/mm^2^, and (**f**) 94 J/mm^2^. Reprinted from Nartu et al. [124], with permission from Elsevier^®^.

The fabrication of Fe–49%Co–2%V alloys was performed by Nartu et al. [124] using a laser-engineered net shaping (LENS) system. Figure 13 presents the distinct microhardness values achieved in the samples of this study, along with the Inverse Pole Figure (IPF) maps and their relationship with grain size. It is shown that an increase in laser power at both scanning speeds leads to a reduction in microhardness. Through the EBSD results derived from the IPF maps, the authors observe that the increase in grain size is responsible for the microhardness behavior. As the laser power increases from 200 W (189 J/mm^2^) to 400 W (94 J/mm^2^) at both scanning speeds, the grain size varies from 52 to 74 μm. The samples underwent two types of heat treatment: (1) A single-step annealing at 950 °C for 30 min followed by water quenching; and (2) a two-step treatment consisting of a first step at 950 °C for 30 min followed by rapid water quenching, followed by a second step at 500 °C for 50 h followed by rapid water quenching.

It was found that the single-step heat treatment promotes a refined grain size due to grain recovery and recrystallization of the BCC grains, resulting in increased H_C_ values. The reduction in H_C_ observed in the two-step treatment led the researchers to perform a TEM study. In this study, they mention that in the two-step treatment condition, substantially larger ordered B2 domains exist, separated by antiphase domain (APD) boundaries. Conversely, in the single-step treatment condition, the samples exhibit weakly ordered nanoscale B2 domains homogeneously distributed within a disordered BCC matrix.

The influence of AM process parameters on the microstructure and atomic ordering of Fe–Co alloys is extensively addressed in the works of Kustas et al. [118,125]. The authors utilized the LENS technique to process Fe–Co–1.5%V alloy, known for its poor workability. One of the investigations [118] provides a detailed characterization of the correlation between the manufacturing process, microstructure, and magnetic properties of the alloy. In the initial study, they explored a range of laser powers from 150 to 450 W, scanning speeds from 2.5 to 10 mm/s, and inter-layer time intervals (t_l_) from 0.4 to 11 s. The microstructural characterization of the as-built samples revealed a fine, equiaxed grain morphology contrary to the expected columnar grain structure. The authors suggest several theories for this microstructural behavior: (1) The repetitive layer-by-layer heating process reduces thermal gradients in subsequent layers, promoting equiaxed solidification instead of columnar. (2) The refinement could be attributed to the inherent phase transformations of Fe–Co alloys during repetitive heating and cooling. The rapid cooling along the γ/α phase boundary prompts the nucleation of a large number of α-BCC grains from the parent γ-FCC solidification structure. This process produces finely equiaxed grains. (3) The presence of oxide particles in the microstructures, which would increase the number of heterogeneous nucleation sites during solidification. However, it is mentioned that this microstructure, based on its grain size and texture, could promote higher creep resistance (via Hall–Petch strengthening), improving the machinability of these alloys and enhancing their magnetic performance. The microstructure of the specimens after annealing at 840 °C for 2 h under vacuum consists of a bimodal, equiaxed, and coarse-grained morphology. The results of the atomic ordering analysis indicate that when a t_l_ of 0.4 s is employed, the relative ordering value is 65 ± 3.2%, while with a t_l_ of 11 s, the ordering value is 43 ± 3.8%. The latter t_l_ value corresponds to faster cooling rates and higher thermal gradients. Therefore, at shorter layer intervals (0.4 s), the high value of the atomic ordering parameter is due to increased heat retention and slower cooling rates during the process. A more detailed analysis of this behavior can be found in [125], where they investigated the formation of B2-ordered phases in Fe–Co–1.5%V alloys, continuing with the use of the LENS process. The aim of this work was to suppress atomic ordering to tailor the microstructure for electromagnetic applications. They employed process parameters that allowed for cooling rates in the range of 10^3^–10^4^ °C/s. This is because cooling rates are typically suitable for suppressing the disorder-to-order phase transformation in conventionally processed Fe–Co alloys. Through EBSD, it is clear that the processed samples have a homogeneous and fine-grained structure with a predominantly equiaxed morphology. The analysis of ordering as a function of cooling rate indicates that the ordering range decreases from 0.72 ± 0.028 to 0.49 ± 0.029 as the cooling rate increases from 1875 ± 282 °C/s to 5764 ± 628 °C/s. However, more detailed analysis results show that a fraction of the ordered B2 phase is still present under conditions where a completely disordered BCC phase is expected. The authors suggest that the reheating of the material in previously solidified layers at high temperatures and the high atomic mobility promote B2 ordering.

The results of these studies reveal that, through the analysis and optimization of AM process parameters, it is feasible to discern their impact on the microstructure of Fe–Co alloys and their influence on magnetic properties. Moreover, alterations in chemical composition and the inclusion of elements like niobium provide insights into controlling atomic structure. This holds potential value not only for these specific systems of soft magnetic alloys but also for other analogous alloy systems.

## 6. Soft Magnetic Composites (SMCs)

The significant advancements in materials science over the past decades, particularly in the control and manipulation of chemical composition, the feasibility of operating at nanoscales, and progress in processing techniques, have led to the emergence of soft magnetic composites (SMCs) [34,35]. These alloys consist of micrometer- or even nanometer-sized particles of iron, Fe–Si, and Fe–Co, among others, coated with an insulating material and are typically consolidated through high-pressure processes. These materials are considered essential in the fabrication of electrical cores, sensors, and other energy conversion devices. This significance is due to their useful properties, such as low core loss, high saturation magnetization, and high electrical resistivity. These properties are directly related to their unique construction. The insulating coating on the powder particles allows for reduced losses from induced currents by increasing electrical resistance. Moreover, the compressed particles maintain tiny air gaps, resulting in increased resistivity [149,150,151].

Additional benefits of SMCs arise from their processing using techniques such as additive manufacturing. The flexibility to shape complex geometries greatly expands their potential applications in the mentioned components [50,111]. A brief overview of various works on AM in these materials is described below.

Borkar et al. [126] processed a Fe–Si–B–Nb–Cu alloy using laser additive manufacturing, varying the proportion of Si and B. Microstructural analysis revealed that when employing low contents of these elements, the microstructure consisted of dendritic α-Fe_3_Si grains. The presence of B and Nb in the interdendritic regions promoted the formation of Fe_3_B grains. As the percentages of B and Si increased, Fe_3_B grains were not observed. However, a eutectic phase enriched in Fe and Nb was formed alongside α-Fe_3_Si grains.

Gheiratmand et al. [52] used the Spark Plasma Sintering (SPS) process to develop the FINEMET^®^ alloy samples (Fe_73.5_Si_13.5_B_9_Nb_3_Cu_1_). These samples are characterized by the random distribution of FeSi nanograins in an amorphous matrix, which enables their attractive soft magnetic properties such as high magnetization, low coercivity, and high Curie temperature. Based on microstructural analysis, the authors demonstrated the presence of the FeSi phase in the amorphous matrix with a grain size of 9 nm and an 84 wt.%. The evaluation of magnetic properties indicated magnetization values of 122.29 emu/g and coercivity of 123 A/m.

Studies conducted by Conteri et al. [127] involving the processing of a Fe_73_._5_Si _13_._5_B_9_Nb_3_Cu_1_ alloy using the LENS process revealed its semi-hard magnetic properties with good saturation magnetization and coercivity. The microstructure consisted of dendritic α-Fe_3_Si grains, with B and Nb in the interdendritic regions forming Fe_3_B grains, along with the presence of Fe_23_B_6_ grains at the α-Fe_3_Si/Fe_3_B interface. The results indicated that the scan speed substantially influenced grain size control.

The continued advancement of these materials and additive manufacturing will continue to contribute to increased efficiency in the components where they are applied. Additionally, the use of these materials based on the utilization of nanoparticles would further enhance their magnetic performance. By reducing the particle size, the losses due to eddy currents can be minimized to the point of being negligible. This highlights the potential for achieving optimal magnetic properties and improved overall performance in various applications [128,129,130,131].

## 7. Soft Ferrites

Soft magnetic ferrites, developed in the 1940s by J.L. Noek [152], are predominantly ceramic materials composed of Fe_3_O_4_ and other elements such as Ni-Zn, Mn-Zn, or Co-Zn. These materials exhibit useful magnetic properties suitable for various high-frequency applications (e.g., transformer cores, inductive toroidal cores in antennae, and different microwave components) due to their high resistivity and low magnetic saturation. Despite the benefits, their processability can be challenging due to their fragility, difficulty in achieving complex shapes, dimensional tolerances, and limitations in size and density [132,133]. However, advancements in additive manufacturing have opened possibilities for their easy processing and subsequent use in electric machines. This can be observed in the studies conducted by Liu et al. [134,135], who developed a UV-curable Ni–Zn paste and fabricated components using a direct-extrusion 3D printer, exhibiting, in some situations, magnetic properties (relative permeability ranging from 63 to 103 and resonance frequency exceeding 30 MHz) similar to those of commercial Ni–Zn ferrite cores. Andrews et al. [136] developed the toroid with Ni–Zn–Cu/Fe–oxide ceramic powder using L-PBF and achieved high permeability values in the fabricated samples. Additionally, the study determined that the proper selection of parameters (process and powder fabrication), as well as heat treatment and weight percentage of ferrite loading, are crucial for reducing porosity and improving magnetic properties.

## 8. Novel Materials: High-Entropy Alloys (HEAs)

As seen so far, soft magnetic materials are essentially composed of two chemical components (e.g., Fe–Si, Fe–Co, or Fe–Ni), which, based on their microstructure, allow them to obtain the previously described magnetic properties for use in electrical devices. However, the constant advancement in materials science and engineering has led to the development of materials based on a greater number of chemical elements, such as high-entropy alloys (HEAs) [153,154]. These alloys exhibit unique characteristics that distinguish them from traditional alloys. They possess a unique crystal structure, which can be body-centered cubic (BCC), face-centered cubic (FCC), hexagonal close-packed (HCP), or combinations thereof. By varying their composition, grain size, and precipitate density, among others, they can exhibit remarkable properties such as high corrosion and wear resistance, high strength, and ductility even at elevated temperatures [155,156,157,158]. Furthermore, these alloys stand out for their ability to vary their chemical composition and adapt to processing conditions, positioning them as magnetic materials with wide application potential. These conditions allow HEAs to exhibit characteristics of both hard and soft magnetic materials, which gives them a promising future in the field of manufacturing motor cores, transformers, power converters, sensors, information system components, etc. [29,159,160,161]. In this regard, the addition of elements with a high magnetic moment, such as Fe, Si, Ni, and/or Co, and the resulting changes they can induce in the distortion of their crystal lattice, improve electrical resistivity and reduce losses due to eddy currents. This renders HEAs candidates for novel soft magnetic materials [161,162,163]. Conversely, the addition of elements such as Al, Ga, Nd, Ti, etc. to these alloys promotes a hard magnetic behavior [159,164,165]. In this section, given the research theme, we will focus solely on the processing of HEAs as materials with soft magnetic properties.

Radhakrishnan et al. [166] investigated the influence of varying Cr content on the soft magnetic properties of CoCr_X_FeNi alloy, where x indicates the atomic percentage of Cr (0–24%), fabricated using laser-directed energy deposition (L-DED). The results of the evaluation of magnetic properties indicate that saturation magnetization and Curie temperature decrease as the percentage of Cr increases. At the same time, coercivity showed values in the range of 1.3–2 Oe.

Zhou et al. [167] processed a FeCoCrNiC_0_._05_ HEA using the SLM process with a laser power of 200–400 W and different laser scanning speeds (800, 1200, 1600, and 2000 mm/s). The evaluation of density indicates that at 800 mm/s and 200 W, the relative density has a value of 99%, while at high scanning speeds and low power, the values of relative density oscillate between 87 and 92%, which increases as the power increases. The effect of these parameters is also observed in the grain size. When the laser power increases from 250 W to 400 W, the grain size increases from 42 μm to 50 μm. On the other hand, the addition of carbon to the alloy indicates that the samples containing C and higher density had a yield strength of 650 MPa and a total elongation of 13.5%. Similar studies were conducted by Park et al. [168], who investigated the mechanical behavior of a CoCrFeMnNi alloy with the addition of 1% carbon using the SLM process. The results demonstrate that SLM enables the attainment of superior tensile properties in this alloy. This is attributed to multiple reinforcing mechanisms such as solid solution strengthening, grain refinement, increased dislocation density, and nanoprecipitate formation. These research efforts highlight the dependence of the microstructure and mechanical properties of HEAs on SLM process conditions, providing valuable insights for optimizing the additive manufacturing of HEAs.

Nartu et al. [169] conducted a comparative study on the mechanical and magnetic properties of CoFeNi alloys processed via laser-engineered net shaping (LENS) and conventionally processed (90% cold-rolled and 90% cold-rolled plus annealed) cast alloys. The LENS-processed sample exhibited a significantly larger grain size (99 μm) compared to the cold-rolled and annealed cast sample (16 μm). Furthermore, the LENS-manufactured sample demonstrated optimal saturation magnetization and coercivity, although its permeability was lower than that of its cast counterpart. On the other hand, severe plastic deformation from the 90% cold rolling of the cast alloy did not significantly affect the Ms and Hc values.

In another study, Yang et al. [170] utilized laser additive manufacturing (LAM) to process an alloy (Fe_60_Co_35_Ni_5_)_78_Si_6_B_12_Cu_1_Mo_3_ using different laser powers (2000, 2500, and 3000 W). Microstructural analysis revealed that the samples consisted of columnar and equiaxed dendrites of the α-Fe–(Co, Ni, Si) phase, along with the presence of secondary phases (Fe_3_B) due to increased diffusion during multiple reheating cycles, resulting in enhanced magnetocrystalline anisotropy and eventual magnetic hardening. The higher saturation magnetization (179–199 emu/g) was observed with increasing laser power, combined with the addition of Cr and increased Fe–Si phase content.

## 9. Summary and Outlook

In this paper, recent advances in the processing of soft magnetic materials through additive manufacturing processes are reviewed. The diversity of materials available for each industry’s demand and their constant development through variations in chemical composition, manufacturing, and post-processing, among others, are noteworthy. The impossibility of having a single material that encompasses all the requirements of an electrical machine compels scientists and engineers to design materials that are adaptable to industrial needs and/or select from those available, justifying their cost based on performance.

Despite existing since the 1940s, soft ferrites are continuously being adapted to current industry demands through new processing approaches, as they are an excellent choice for applications where high power densities are not required. Soft magnetic composites (SMCs) and high-entropy alloys (HEAs) with soft magnetic properties are shown to be the most promising materials in this field for use in electrical machines. This is due to their remarkable properties, such as good deformability, high-temperature mechanical strength, fatigue, and corrosion resistance, which are a result of the combination of chemical elements, grain size modification, precipitate formation, and grain orientation, among others. Additionally, their unique nanostructure and the ability to work with extremely thin laminations and geometric variations have allowed for low energy losses due to eddy currents while maintaining suitable M_s_ values. However, Fe–Si, Fe–Ni, and Fe–Co alloys remain the most widely used materials in these applications, as they combine notable magnetic properties such as high resistivity, permeability, magnetic saturation, and low energy loss. Their microstructural characteristics, mechanical properties, and technological impact have been extensively studied, leading to continuous innovation and development. Among these materials, Fe–Si alloys are the most commercially affordable and widely available, making them highly suitable options for electrical machines.

The constant evolution of these materials is a reality that, based on improved control over the microstructure and chemical composition of existing and emerging materials, has allowed for increased magnetic properties. This review shows that optimizing the structure within magnetic materials will require greater use of advanced characterization tools, such as electron microscopy, X-ray characterization, and magnetic force microscopy, among others. In addition to material optimization, ongoing activities in research groups worldwide indicate that additive manufacturing is a viable manufacturing process for developing components based on these materials while maintaining or enhancing their magnetic properties and reducing processing costs. Many soft magnetic materials, such as Fe–Ni, Fe–Co, Fe–Si, and SMCs, have been successfully fabricated using additive manufacturing, particularly with processes like L-PBF, SLM, DED, and LENS. The effect of process parameters, such as laser speed, laser power, printing direction, and subsequent heat treatment, has been discussed as determining factors for the microstructure, mechanical properties, and magnetic properties of the final product.

Furthermore, additive manufacturing has enabled the implementation of novel and radical design concepts for magnetic components, allowing for the spread of SMMs in applications where their use was previously limited. The comprehensive review in this work provides interesting data that could contribute to the future of AM technology. This review illustrated the need for the development of other AM components in the future, such as thermal sensors capable of providing on-site diagnostics during printing, temperature-controlled construction platforms to manage thermal gradients during printing, and high-temperature ovens for automated post-printing heat treatments.

Finally, studying and presenting the various advancements and discoveries in soft magnetic materials and their application in additive manufacturing to the scientific community allowed for contrasting research results and identifying different practices, techniques, and promising materials for use in magnetic components, electric motors, transformers, sensors, etc. Moreover, this work can serve as an analytical point to identify knowledge gaps and areas for future research in this field, guiding researchers toward areas that require more effort and where significant advancements can be achieved. It is now the responsibility of the community of scientists, engineers, and other professionals to enhance these materials and their processing techniques through scientific knowledge to meet the requirements of the next generation of electric machines.

## Figures and Tables

**Table 1:** List of the general values of the main magnetic properties of SMMs utilized in the reviewed articles, together with the applied AM technique.

SMMs		Saturation Magnetization M_S_ (T)	Relative Permeability μ_r_	Core Resistivity **r (μΩcm)**	AM	Ref.
Fe–Si	Fe–(2.9–3.7) Si	2.10	25	20–50	L-PBF, SLM	[87,88,89,90,91,92,93,94]
	Fe–(6.5–6.9) Si	1.9	11,000	85		[95,96,97,98,99,100,101,102,103,104,105]
Fe–Ni		0.60–1.10	8000–100,000	-	L-PBF, SLM, DED	[106,107,108,109,110,111,112,113,114,115,116,117]
Fe–Co		2.4–2.5	20,000–60,000	45	L-PBF, LENS	[118,119,120,121,122,123,124,125]
SMCs		0.50–1.30	6000–100,000	120–130	SLM, DED, SPS	[52,126,127,128,129,130,131]
Soft Ferrites		0.32–0.545	350–20,000	10^7^ × 10^9^	L-PBF, DED	[132,133,134,135,136]

## Abbreviations

AM	Additive Manufacturing
APD	Antiphase Domain
B	Flux Density
BCC	Body-centered Cubic
BJP	Binder Jetting Technology
B_r_	Remanence
CAD	Computed-Aided Design
DED	Directed Energy Deposition
E	Specific Energy/Specific Laser Energy
EDS	Energy-dispersive X-ray Spectroscopy
EBSD	Electron Backscatter Diffraction
E_V_	Input laser energy densities
FCC	Face Centered Cubic
H_C_	Coercivity
HCP	Hexagonal Close-packed
HEAs	High-entropy Alloys
HIPing	Hot Isostatic Pressing
HT	Heat Treatment
HV	Hardness Vickers
IPF	Inverse Pole Figures
k	Thermal Conductivity
LBM	Laser Beam Melting
L-DED	Laser-directed Energy Deposition
LENS	Laser-engineered Net Shaping
L-PBF	Laser Powder Bed Fusion
MIM	Metal Injection Molding
M_r_	Relative Permeability
MRI	Magnetic Resonance Imaging
Ms or Bs	Saturation Magnetization
r	Electrical Resistivity
SEM	Scanning Electron Microscopy
SLM	Selective Laser Melting
SMCs	Soft Magnetic Composites
SMMs	Soft Magnetic Materials
SPS	Spark Plasma Sintering
TEM	Transmission electron microscopy
TO	Topological Optimization
XCT	X-ray computed tomography
X_m_	Magnetic Susceptibility
XRD	X-ray Diffraction
